# Image registration in dynamic renal MRI—current status and prospects

**DOI:** 10.1007/s10334-019-00782-y

**Published:** 2019-10-09

**Authors:** Frank G. Zöllner, Amira Šerifović-Trbalić, Gordian Kabelitz, Marek Kociński, Andrzej Materka, Peter Rogelj

**Affiliations:** 1 Computer Assisted Clinical Medicine, Medical Faculty Mannheim, Heidelberg University, Theodor-Kutzer-Ufer 1-3, 68167 Mannheim, Germany; 2 Faculty of Electrical Engineering, University of Tuzla, Tuzla, Bosnia and Herzegovina; 3 Institute of Electronics, Lodz University of Technology, Lodz, Poland; 4 Faculty of Mathematics, Natural Sciences and Information Technologies, University of Primorska, Koper, Slovenia

**Keywords:** Kidney disease, Image registration, Dynamic MRI, DCE-MRI, ASL

## Abstract

Magnetic resonance imaging (MRI) modalities have achieved an increasingly important role in the clinical work-up of chronic kidney diseases (CKD). This comprises among others assessment of hemodynamic parameters by arterial spin labeling (ASL) or dynamic contrast-enhanced (DCE-) MRI. Especially in the latter, images or volumes of the kidney are acquired over time for up to several minutes. Therefore, they are hampered by motion, e.g., by pulsation, peristaltic, or breathing motion. This motion can hinder subsequent image analysis to estimate hemodynamic parameters like renal blood flow or glomerular filtration rate (GFR). To overcome motion artifacts in time-resolved renal MRI, a wide range of strategies have been proposed. Renal image registration approaches could be grouped into (1) image acquisition techniques, (2) post-processing methods, or (3) a combination of image acquisition and post-processing approaches. Despite decades of progress, the translation in clinical practice is still missing. The aim of the present article is to discuss the existing literature on renal image registration techniques and show today’s limitations of the proposed techniques that hinder clinical translation. This paper includes transformation, criterion function, and search types as traditional components and emerging registration technologies based on deep learning. The current trend points towards faster registrations and more accurate results. However, a standardized evaluation of image registration in renal MRI is still missing.

## Introduction

Magnetic resonance imaging (MRI) modalities have achieved an increasingly important role in the clinical work-up of chronic kidney diseases (CKD) [1]. They allow a minimal-invasive measurement of a panel of parameters that can play an important step for the diagnosis and monitoring of renal diseases. This comprises among others assessment of kidney volumes [2], microstructure via diffusion weighted imaging [3], hemodynamic parameters by arterial spin labeling (ASL) [4], or dynamic contrast-enhanced (DCE-) MRI [5]. Especially in the latter, images or volumes of the kidney are acquired over time for up to several minutes. Therefore, they are hampered by motion, e.g., by pulsation, peristaltic, or breathing motion. This motion can hinder subsequent image analysis to estimate hemodynamic parameters like renal blood flow or glomerular filtration rate.

Image registration in the context of renal imaging in this review is the spatial alignment of intra-subject kidneys taken in a certain time range (a single imaging session) to improve further processing steps for an improvement of the image analysis. Some techniques allow inter-subject image registration to deploy generalized models to advance diagnosis.

Barriers in the development of renal MRI biomarkers with respect to CKD are, among others, the limited availability of software tools to analyze and extract the renal data. Furthermore, the lack of access to data from previous studies hinders the development benchmarks for such tools.

This is not only limited to renal image registration, but is a limiting factor in medical image registration in general. In a recent review, Viergever et al. [6] list of comments and observations include the “emerging need of public databases of representative expert-annotated images and of validation protocol” and “the rare use of registration in diagnostic clinical practice”.

To overcome motion artifacts in time-resolved renal MRI, motion correction strategies have been proposed for over a decade; however, its translation into clinical practice is still missing. Renal image registration approaches could be grouped into (1) image acquisition techniques, (2) post-processing methods, or (3) a combination of image acquisition and post-processing approaches.

The Working Group 2 of the COST (European Cooperation in Science and Technology) action PARENCHIMA (Magnetic Resonance Imaging Biomarkers for Chronic Kidney Disease) (http://www.renalmri.org) investigates renal data analysis algorithms including image registration to provide a core software library for a comprehensive and standardized approach to renal data analysis. The aim of the present article is to discuss the existing literature on renal image registration techniques and show today’s limitations of the proposed techniques that hinder clinical translation.

## Image registration techniques

### Image acquisition based

Organ motion in (renal) MRI causes image artifacts due to displacement during the quiescent period between each data sampling period and the following excitation radio frequency (RF), and as a result of spin phase induced by motion through magnetic field gradients between an excitation RF pulse and the subsequent data sampling period. To correct these during the image acquisition, three general approaches can be followed: (1) breathhold strategies reducing the amount of movement, (2) using a navigator echo pro- and retrospectively trigger the acquisitions, and (3) image readouts that suppress motion artifacts. Recent work reporting approaches in any of the three categories is summarized in Table 1.

**Table 1:** Overview of image acquisition-based motion correction techniques used in renal MRI

	Motion correction approach	Application	References
Breathhold strategies	Shallow regular breathing	ASL, DCE-MRI	Brox et al. [7]
			Schewzow et al. [46]
	Breathhold during first pass of contrast agent	DCE-MRI	Melbourne et al. [25]
	Repeated breathholds	ASL, DCE-MRI	Eikefjord et al. [10]
			Robson et al. [8]
			Schewzow et al. [46]
Pro- and retrospectively trigger	Retrospective gating	DCE-MRI	Attenberger et al. [9]
Image readouts	Radial readout (KWIC)	DCE-MRI	Eikefjord et al. [10]
	Propeller	DCE-MRI	Lietzmann et al. [12, 13]
	Radial readout scheme, golden-angle increment and iterative reconstruction	DCE-MRI	Riffel et al. [14]
			Kurugol et al. [15]
	Keyhole + compressed sensing	ASL	Taso et al. [16]

In renal perfusion MRI, several approaches use breathhold techniques to mitigate kidney movement. These range from shallow regular breathing [7], breathhold during the first pass of contrast agent in DCE-MRI to repeated breathholds as for instance in ASL [8]. For subsequent data analysis like perfusion quantification interpolation or image-based post-processing, image registration is used.

Navigator echoes are extra data acquisitions capturing motion or motion-related change of phase. Similar to external triggering of respiratory motion, these data can be used to either trigger data acquisitions prospectively, i.e., only data in a certain respiratory phase are accepted or to record the breathing pattern and discard those data not within the capture window retrospectively. Prospectively gated acquisitions usually take longer to acquire the amount of data needed and are usually not used in dynamic imaging as the data sampling is hard to control. Retrospectively gating of renal perfusion MRI is feasible if the data are acquired at high temporal rate, such that the remaining accepted data sufficiently capture the signal change over time as shown by Attenberger et al. [9].

Center-out imaging readouts such as projection reconstruction or radial and spiral MRI have been shown to reduce motion artifacts. This is attributable in part to oversampling of central k-space, which reduces artifacts in a manner similar to multiple averaging in conventional imaging. In addition, when the data collection begins at the center of k-space, in-plane gradient moments are reduced in the central region of k-space, minimizing spin phased induced motion artifacts. In renal DCE-MRI, Eikefjord et al. [10] compared a radial readout scheme [k-space weighted image contrast (KWIC) filter] to a Cartesian sampling technique [Fast Low-Angle Shot (FLASH)]. Patients were instructed for repeated breathhold during the whole acquisition. Results showed that using the same post-processing scheme and pharmacokinetic model, FLASH produced more accurate perfusion and filtration parameters than KWIC did compared with clinical reference methods.

A combination of oversampling the k-space center and navigator information has been proposed by James Pipe, called Periodically Rotated Overlapping ParallEL Lines with Enhanced Reconstruction (PROPELLER) MRI [11]. In this, a small strip of several rectangular samples’ k-space lines is acquired and then consecutively rotated around the k-space center. The latter aspect of PROPELLER MRI permits correction for in-plane displacement and rotation, i.e., patient motion, image phase due to motion, and through-plane motion.

This approach has been extended by Lietzmann et al. [12] for 2D DCE-MRI. This sequence was parameterized according to a T1-weighting using a saturation prepulse. Different parametrization of the PROPELLER readout was tested. Compared to a widely used TurboFlash DCE-MRI exam, reduced motion artifacts could be obtained [13].

Recently, a combination of radial readout scheme, golden-angle increment, and iterative reconstruction called GRASP was proposed for dynamic kidney imaging by Riffel et al. [14]. The latter allows for reconstructing image dynamics at different temporal resolution featuring high-resolution morphological images or high temporal dynamic images by including different amounts of the acquired radial spokes. Riffel et al. demonstrated that a high overall image quality score for the best arterial phase and the best renal phase and a high diagnostic confidence in the obtained images could be obtained. Minimizing residual undersampling artifacts could be reached utilizing 55 spokes, while a high temporal resolution is achieved by 13 spokes. There were no respiratory motion artifacts in any of the patients. Streak artifacts were present in all the patients, but as compared to the KWIC did not compromise diagnostic image quality. The estimated renal blood flow (RBF) was slightly higher (295 ± 78 mL/100 mL/min) than reported in previous MRI-based studies, but also closer to the physiologically expected value.

A similar approach to Riffel et al. was demonstrated in a pediatric population using a radial volume interpolated breathhold exam (VIBE) sequence and the GRASP reconstruction framework by Kurugol et al. to estimate single kidney GFR [15].

Combining a keyhole technique with compressed sensing (CS) image reconstruction in pseudo-continuous ASL was recently proposed by Taso et al. [16]. In their work, ASL preparation was combined with a variable-density (VD) elliptic Poisson-disk segmented Cartesian fast spin-echo (FSE) readout including a fully sampled 6 × 6 k-space center region. To provide motion robustness, the outer k-space was pseudo-randomly undersampled to increase the temporal resolution. To reach an overall k-space sampling, the undersampling scheme was changed between repetitions. The authors designed a sampling enabling a minimum of three shots for each individual volumetric repetition because of resolution, slices, and echo train-length constraints. This acquisition was then repeated multiple times with variable outer k-space sampling. While each repetition was accelerated up to *R* ≈ 23, the overall k-space coverage led to an effective acceleration of *R* = 3.8. The acquired raw k-space data were offline reconstructed using a 4D k-t-CS parallel imaging using eigenvalue maps (ESPIRiT) reconstruction [17]. In three healthy volunteers, Taso et al. demonstrated that whole kidneys’ isotropic free-breathing perfusion measurement using ASL is feasible in about 5 min with image quality comparable to a single-slice single-shot fast spin-echo (SSFSE) and post-processing motion correction.

### Image post-processing-based

Image post-processing-based renal MRI registration methods differ according to three key components of image registration techniques, i.e., criterion function, geometric transformation model, and search method. An overview of image registration techniques in renal MRI is given in Table 2.

**Table 2:** Overview of image post-processing registration techniques according to the two key components, the criterion function and the geometric transformation model

	Criterion function			
	Intensity-based	Edge- or gradient-based	Fourier transform	Intensity correction model
Geometric model				
Translation	Brox et al. [7]	Zikic et al. [30]	Giele et al. [39]	Buonaccorsi et al. [35–37]
Rigid	Rogelj et al. [26]	de Senneville et al. [40]	Song et al. [32]	
	Positano et al. [20]	Yim et al. [33]		
	Fei et al. [24]	Haber et al. [107]		
	Brox et al. [7]	Sun et al. [31]		
Deformable	Zöllner et al. [21]			
B-splines	Brox et al. [7]			
	Anderlik et al. [23]			
	Tokuda et al. [79]			
	Sance et al. [22]			
	Melbourne et al. [25]			
Deformable non-parametric	Zöllner et al. [21]	Hodneland et al. [18, 19]		Lausch et al. [29]
	Rogelj et al. [26]	Eikefjord et al. [10]		
	Merrem et al. [28]			

#### Objective function

Selection of an objective function (also known as cost function or loss function) seems to be the most challenging decision at implementation of renal registration algorithms. Intensity values at the same anatomical points may differ considerably for images acquired under different conditions, e.g., presence of contrast agent in DCE-MRI or magnetization of the inflowing blood in ASL. Thus, subsequent images do not differ only due to kidney motion that need to be corrected, but also due to beneficial information that needs to be preserved. The most common approach to define an objective function is to select an intensity-based similarity metric that best measures alignment of two images. Due to image differences, the far most widely selected measures are mutual information (MI) [7, 18–23] and normalized mutual information (NMI) [24, 25]. Other statistically based similarity measures are rarely used, e.g., point similarity measures that build on top of MI [21, 26]. The correlation ratio that assumes functional intensity dependence [27] or cross-correlation [28] is also applicable.

In contrast to image intensity values, image gradients are expected to be less dependent on the expected beneficial image differences. Consequently, several groups have developed algorithms to use gradient information instead of pure intensity information. Not only the magnitude but also the direction is used in normalized gradient field (NGF) proposed by Haber and Modersitzki which is applied by several other groups [10, 18, 19, 29, 30]. Similar to this is the edge-based consistency metric [31].

Others have split objective function computation in a two-step process, where the first step is image preprocessing that extracts image features relevant to kidney motion and the other step is measurement of similarity, using mono-modality measures. Such example focused on image gradient information was presented by Song et al. [32]. They have extracted image edges using wavelet edge detection and further registered images using a Fourier transform-based registration approach.

In some cases, edge information is used only to detect the kidney region, to which the search of deformation was limited [24], or only the kidney edge region was used [33].

In contrast, some solutions rely on similar appearance of successive images and use mono-modality similarity measures, e.g., sum of squared differences (SSD), and multiple reference images [34].

A more sophisticated solution is an intensity correction method that reduces image differences not related to kidney motion, proposed by Lausch [29]. Here, the intensity correction reduces effect of different amount of contrast agent in the image volumes, enabling the use of SSD similarity measure. Similarly, a tracer kinetic model-driven registration procedure applies a tracer model to the reference image to equalize its intensities with each of the images in a sequence before registering them [35–37]. Thus, in contrast to using pure intensity-based similarity measures that due to image differences need to have multi-modality capabilities, the criterion function now employs an additional intensity correction model and only a simple mono-modality similarity measure.

To prevent tissues surrounding the kidneys from influencing the registration results, registration methods may limit the estimation of criterion function to a region of interest (ROI). For example, both kidneys move independently which will lead to a lower performance when applying a global rigid registration rather than two ROIs surrounding each kidney which are transformed independently by the registration approach [38]. The definition of an ROI links image registration with image segmentation. Most often, the ROI is defined manually for the reference image as delineation of the renal cortex [10, 31, 39]. If needed for other images as well, the reference ROI may be replicated to other images [20, 40]. The ROI does not always need to tightly match a kidney region. The quality of segmentation does not affect registration result considerably and only an approximate localization of the kidney may be needed [26]. Tissues that closely surround the kidneys are not affected by contrast agent passage and including them in the ROI may actually help the registration process [40]. Often, the defined ROI deliberately expand the kidney region [20], and in some cases, it turns out that it is sufficient to define the ROI as a rectangular region [31].

#### Geometric transformation model

To register one kidney image to the other, the expected geometric changes of the kidney must be modeled by a geometric model. The geometric changes of kidneys have many causes, e.g., breathing, heartbeat, peristalsis, and are extremely difficult to track or to describe them geometrically [37]. The largest estimate of displacements during normal breathing which we have found in the reviewed literature is 7 mm (LR), 20 mm (SI), and 7 mm (AP) [29], while some other estimations are in a similar range [41]. In forced deep breathing translations, up to 86 mm are reported [42]. The deformation component is more difficult to estimate and the extent of expected deformation is currently not clearly evaluated, although it has been shown that the kidney shape variability can be modeled using an elastic model [43] or an active shape model [44]. In clinical practice, it is considered that the extent of deformation is negligible and a rigid model is sufficient for reaching the correct diagnosis [20, 40]. Nevertheless, some experiments show that visually better results may be obtained using non-rigid approaches, although this may not necessary be due to actual kidney deformation but also due to consideration of other image differences that are not anatomical in nature, e.g., movement of the contrast agent.

To select the geometric transformation model, one also needs to consider the fact that information obtained from the images can be insufficient to reliably map each point on one image to the corresponding point on the other image, e.g., deformation of a homogeneous region cannot be quantified. Furthermore, transformation visible on the images is not only due to kidney geometry but also due to other factors, e.g., passage of the contrast agent. Consequently, large intensity variations in perfusion scans can lead to an apparently changed shape of the kidney in the image, which results in errors in the estimation of kidney parameters [30]. Due to this, stronger penalization and restriction of volume change were proposed for images with contrast differences.

Such restrictions of the geometric transformation can be implied by a transformation model and/or by explicit regularization. Low parameter geometric models correspond to smooth image deformation, so that additional regularization is not needed. Among such, the most often used one is a rigid model [24, 32, 41]. Even more limiting is a translational model [30, 31, 35], which is sometimes acceptable due to reduced computational time. The affine transformation model is usually not used, since the kidneys do not undergo shearing or considerable changes in size what would correspond to scaling.

Among non-rigid models, the most commonly used one is a B-spline model [7, 9, 22]. It does not require an additional explicit regularization, because the extent of deformation can be controlled by the density of control points. On the contrary, non-parametric models do need explicit regularization, which may follow the physical laws of elasticity [10, 29, 45] or viscosity or consider other usually simplified dependencies to enforce smoothness, e.g., Gaussian [21, 28]. Smoothness can be imposed not only in the spatial dimensions but also in the temporal dimension [31].

#### Search method

Image registration is a procedure of finding a transformation that complies with the given transformation model and minimizes the given criterion function. This is generally an optimization problem. To increase attraction range, computing efficiency, and reliability of optimization at unavoidable presence of local extrema of criterion functions, the search may hierarchically use images of different resolutions [22, 29, 44] and gradually increase the complexity of transformation model used, from more restrictive rigid ones to more and more detailed deformable ones [22]. It is common that non-rigid registration is preceded by a rigid one [22, 23, 28].

The optimization method is selected depending on the number of transformation parameters. For low number of parameters (rigid, translations only), methods that do not require gradient information are used, e.g., Nelder and Mead simplex method [24, 35] or Powell method [26]. For larger number of parameters, a gradient-based algorithm is used. B-spline transformation models are often optimized using gradient-descent methods [7] or Broyden–Fletcher–Goldfarb–Shanno (BFGS) methods [21, 22]. Non-parametric methods, where the coordinates of all voxels can be considered as parameters, are usually implemented using gradient-descent methods [26, 28], although Newton or quasi-Newton solvers are also applicable, including the BFGS method [29]. If landmarks or easily distinctive points are recognizable, point-based optimization becomes another option. Using popular solver like iterative closest point, Newton’s method or quasi-Newton methods can solve the non-linear optimization [46].

Avoiding the optimization, translations can also be obtained directly by analysis in the frequency domain using Fourier transformation. A phase difference movement detection (PDMD) is shown to be efficient and depends on the phase spectrum only [39]. Using an optimization method, the frequency spectrum also enables to estimate all six rigid parameters, including rotations [32]. Fourier-based approaches are reported to be very sensitive to the determination of the mask with which the surrounding tissues are removed from the images [40].

For 4D sequences, a selection of a reference frame, to which other frames are registered, is important. Often, the selection of the reference needs to be made manually [30], usually selecting the image in which kidney compartments are visible best. Lausch automates the selection by choosing a volume with the maximum average voxel intensity [29], while Merrem et al. select the reference randomly [28]. In contrast to using the same reference frame for all the frames in a sequence, an incremental approach registers only successive frames to each other [39]. An advantage of this approach is that subsequent images have similar contrast and can be registered using mono-modality criterion functions, while the problem is in accumulation of the registration error. The problem can be reduced by increasing the temporal step and registering multiple successive images to the same Ref. [33]. Wright et al. propose another approach in which the top of the liver is detected and used to infer the kidney position [34]. Then, all images at the most frequent position are used as reference for registering other images, such that each image is registered to temporally the nearest reference. Siva et al. oppose this solution by finding out that the magnitude of the kidney motion cannot be reliably estimated from diaphragmatic, liver dome, or abdominal wall surrogates [42].

#### Deep learning approach

In recent years, plenty of papers reporting a use of Deep Learning (DL) for medical image analysis have been published. These approaches have already demonstrated very high potential for efficient medical image processing and analysis [47–50] and are believed to play a significant role in medical registration [6]. Viergever et al. refer to an earlier review on the same topic and show a confrontation between trends noticed 20 years ago and recent developments. Their reach list of comments and observations include the “emerging need of public databases of representative expert-annotated images and of validation protocol” and “the rare use of registration in diagnostic clinical practice”. They expect that the deep learning approaches to registration might dominate the field of image registration; on the condition, the validation protocols and clinical acceptance will be the focus of attention, as well.

An extensive overview on DL in medical image analysis with the focus on MRI [48] contains a short section on DL applications to image registration, and there are 26 papers referred to in that section. The advantages of using DL instead of standard deformable registration algorithms are in accuracy [51] and speed improvements [47]. Application of DL in medical image analysis and image registration is reported in various reviews [47, 49, 50, 52] but little in renal image registration. However, many papers cited contain essential contributions that can be transferred to the kidney image registration task.

In Lv et al., a DL method for abdominal MRI registration is described, aimed to obtain motion-free images throughout the respiratory cycle [53]. In fact, it is a modification of the method of Buerger et al. where a one-dimensional fast Fourier transform was applied along the feet–head direction to the center k-space profiles, to compute the projection profiles of the 3D volume [54]. From the envelope of the projections time-course, the respiratory motion signal was estimated. Based on this signal, near motion-free data were identified and used to reconstruct the high-quality reference images at the end-respiratory acceptance window. The images reconstructed from k-space data taken at other phases of the respiratory cycle were registered to the reference images. Unlike in Buerger et al. where a local adaptive affine registration algorithm (LREG) was used [54], the motion-induced image deformations were corrected with the use of a convolutional neural network. The network takes patches from stationary and moving object image at the same locations and generates two momentum predictions of the patches in the *x-* and *y*-directions. They are used to generate dense displacement vectors interpolated with cubic B-splines. In the training phase, normalized cross-correlation between target and moving image patches was used as the similarity measure in the cost function optimized with the Adam optimizer [55], available in the Tensorflow environment [56]. On a Nvidia GTX 1080 GPU, the training on data from 2.490 448 × 448-pixel images of ten healthy subjects took about 26 h. The elaborated algorithm showed better quality scores compared to the LREG method; both gave better results than the non-motion-corrected method. The CNN-based image reconstruction significantly reduces the registration time, from 1 h (LREG) to 1 min.

In general, there are two subtasks of the image registration pipeline which are solved with the use of deep learning. In both cases, the neural network works in the regression mode. First approach refers to the use of a network for estimation of similarity measures between the two images [57–59]. The estimated differences are then minimized in a traditional registration procedure, e.g., through non-linear optimization of a geometric transform of image coordinates. The similarity measures are learned straight from the image data to represent complex relationship between local intensity distributions of the images, apparently not captured by traditional handcrafted statistical estimators. In the second approach [60, 61], the neural network predicts the parameters of the voxel coordinate transformation, and thus, the time-consuming iterative optimization is eliminated and the registration becomes faster—can be performed in real time. The work of Cao et al. integrates both approaches [62].

Yet, another characteristic of the dynamic kidney registration problem is the contents of images changes in time as the contrast medium (magnetized blood in ASL) dynamically spreads out through the tissues. The image intensity changes caused by kidney anatomical elements displacement due to motion cannot be distinguished from the changes originating in blood perfusion, as explained in the Criterion Function section. As a result, the images which are being matched together become multimodal. The registration of multimodal images poses a known challenge to the image processing community [51]. The two fundamental problems involved in accurate fusion of different modality medical images—definition of representative similarity metrics and efficient optimization algorithms for deformation—were addressed. A deep convolutional neural network (CNN) was proposed to learn the similarity metrics between the magnetic resonance and transrectal ultrasound (TR-US) volumes, used for prostate cancer diagnosis. It is an exceptionally challenging task, as the TR-US and MR volumes occupy different field of views and their intensity distributions lead to substantially different appearance. The inputs to the CNN were image patches and the output—an estimate of their misplacement. The network was trained using images that were manually registered by experts. A Keras–Tensorflow environment [63] was used for training with Adam optimizer, and the loss function was the mean-square error between the CNN-predicted and ground truth registration. Strategies for careful generation of image samples for training and multi-pass optimization for rigid image correction were developed. The method was validated and evaluated on 679 data sets. Superior performance in terms of significantly smaller registration errors as compared to mutual information-based features and to modality-independent neighborhood descriptors [64] was demonstrated. This example is, indeed, encouraging. We believe that the difficult problem of kidney image registration can be effectively solved with CNNs, by making use of their inherent flexibility in acquiring knowledge hidden in images [65].

A very important and practical topic is considered in a study by Tajbakhsh et al. addressing the issue of training deep CNNs for medical image analysis [66]. This operation takes a very long time, especially when undertaken from scratch. Moreover, it requires a large number of training examples, which, in the case of supervised learning, should be labeled what makes the whole process laborious and costly. A very attractive alternative is in CNN re-training—through a shallow or deep fine-tuning. The knowledge incorporated in CNN weights trained on one kind of images can be transferred to a CNN aimed to analyze images of another type. The feasibility of such transfer was demonstrated in some publications devoted to recognition of natural images. In Tajbakhsh et al., it was investigated more thoroughly in the context of medical applications. Four different applications and three imaging modalities were considered. The experiments have shown that knowledge transfer from natural to medical images is possible. The re-trained CNNs were performing better or at least not worse that those trained from scratch. The fine-tuned CNNs were more robust to the training set size. A CNN layer-wise fine-tuning was developed as a practical way to obtain the best CNN performance. These results are very promising; searching for optimal ways of knowledge transfer from pre-trained neural networks is certainly a research direction of high potential.

Another approach to bypass this inaccessibility is the use of synthetic data for the training procedure. In particular, synthetic data can overcome the issues of limited data set size and inaccurate annotations [67, 68]. Recent advances in the field of DL demonstrated comparable results in the case of photon scatter estimation based on digital phantoms [69]. Furthermore, the application of generative adversarial networks (GANs) [70] for the synthesis of photo-realistic retinal images based on morphological data showed convincing results [71]. A step further is the utilization of the so-called cycleGANs (cGANs) [72]. CGANs allow the mapping between two domains given unpaired training samples. Two mapping functions mapping between the two domains are called generators. Two additional networks, called discriminators, aim to distinguish between real images and generated images. Russ et al. recently showed an approach to generate synthetic CT data sets to train a DL network for vessel segmentation using cGANS [73]. Tanner et al. proposed CT to MR image registration using cGANs [74]. Such approaches seem promising to solve shortage of annotated data, also for DL-based image registration.

Deep learning frameworks for deformable unsupervised registration were developed recently for brain MRI [75, 76] as well as cardiac cine MRI and CT chest images [77]. Weakly supervised CNN was proposed for multimodal MR-TRUS image registration [78]. The benefits in higher registration accuracy and reduction of computation time have been clearly demonstrated. Possibility of neural network (NN) transfer learning is an attractive property which should be explored to a larger extent. On the other hand, there is a very limited activity in the area of kidney image registration with the use of deep learning techniques.

### Evaluation

Prior to a clinical application, the medical image registration algorithms for renal MRI need to be carefully validated. Validation of registration accuracy, especially for non-rigid image registration methods, is considered as a non-trivial and difficult task, because the ground truth (i.e., gold standard) is generally not available. The image registration algorithms are aimed towards solving multiple problems that arise during renal image alignment, such as: the rich variety in the anatomy and pathology; the lack of fiducial markers on the kidneys; the change in the intensity in a MR image during data acquisition; the variability in kidney motion and geometry in MRI images, and the lack of standard data sets. These problems make it very hard to evaluate the accuracy of the registration methods and require carefully studied validation protocols. To assess the registration accuracy, several strategies have been proposed. Evaluation of registration approaches often relies on the visual inspection by an expert user, or a controlled study using computer simulations or physical phantoms.

Visual inspection is often used for the evaluation of the image registration quality [41], but it requires a special expertise and extensive experience. A color overlay of registered image has been often used as a way to assess the registration quality [24]. The quality of the registration has also been assessed by visual inspection of checkerboard images compiled from fixed images and moving images [7, 22, 23, 26, 28, 46]. A checkerboard image is obtained by patching one square region from the fixed image and another square region from the moving image after registration, and these patches are visualized in one checkerboard image. If two images are correctly registered, the contours of the kidneys and other structures should be aligned, and should show continuously lines, while the disparities between these two images indicate errors. In Zikic et al., visual result is confirmed by analyzing the frequency spectrum of the signal [30].

Assessment of registration performance in DCE-MRI motion correction has been also addressed using pharmacokinetic modeling [25, 29, 79]. The goodness-of-fit and smoothness of time-curves are frequently used criteria for successful registration evaluation of DCE-MRI data [18]. The goodness-of-fit of the time series to a pharmacokinetic model is expected to be high if the data are smooth and, therefore, can be easier fitted by the fitting algorithm. Smooth time series data are achieved by a good image registration. Therefore, in these approaches, the registration accuracy is assumed to be coupled to the model fitting and the goodness-of-fit can be used as image registration evaluation criteria, respectively. Deviation of estimated voxel-wise GFR values of the motion-corrected time-courses of DCE-MRI of the kidney from Iohexol measurements has been also used as an evaluation criterion [18]. In de Senneville et al., the evaluation is based on the improvement after movement correction using the Patlak–Rutland model [40].

Another applied approach to validate registration results is by generating realistic synthetic phantom data sets used as a ground truth [32, 35]. In Buonaccorsi et al., for instance, a procedure for tracer kinetic model-driven registration for DCE-MRI time series data is described and validated against a software phantom data set that is set to mimic a full DCE-MRI image set [35].

Some authors reported an evaluation based on the coronal motion: the deviation of the vertical position of the kidney [12, 13, 28]. They measured and reported a reduction of coronal motion after a deformable registration. Similar, in Boer et al., as a measure of respiratory induced motion, root-mean-square (RMS) vertical misalignment of the top of the kidney was measured manually with respect to the first time point on all recorded volumes [80]. As another measure of registration error, the whole parenchyma time–intensity curve was calculated on all images using fat images. In Giele et al., the performance of the movement correction is reviewed by an operator, and his manual adjustments were recorded and compared to the proposed method [39]. However, the aforementioned methods measured registration performance in one direction only, assuming that the horizontal movements were minor.

The mean intensity curves of carefully selected ROIs and combined with standard deviations have been used for an assessment of motion correction [7, 22, 26, 32], but usually, these items are just indicative and not absolute. In addition, in Zöllner et al. obtained variances within the selected ROIs were analyzed by the F test to investigate whether there are significant differences between the registered and unregistered data [21].

In several papers, image quality and artifacts are estimated by the expert readers [14, 39, 81]. The registration algorithm was validated against manual registration or segmentation performed by an expert user [20, 32, 82]. The target registration error (TRE) can be determined by the Jaccard or Dice coefficient measuring the overlap between the manual segmented target object. This still requires a segmentation of the reference and template image [83]. Another evaluation approach includes the distance measures, like Hausdorff distance, of distinctive landmarks of the kidney [84]. The landmarks can be acquired manually or automatically.

The movement correction by image registration in ASL techniques in the kidney is assessed through the subsequent perfusion rates quantification [41]. The authors measured the reduction in the estimated medulla perfusion rate before and after image realignments. Similarly, evaluation of the registration method for the application of quantitative analysis of kinetic parameters is performed by estimation of the perfusion and filtration parameters on the original input data and the same series after motion correction [30].

Some authors are applying landmarks to analyze the motion occurring in images before and after their registration. In Gupta et al., the anterior right ventricular (RV) insertion point was manually identified as a landmark, in each image [85]. Accuracy of registration was quantified by measuring the motion of this landmark between successive image pairs, in both unregistered and registered time series.

An evaluation of the registration accuracy by fitting a two-compartment model to data (before and after registration) and calculating Akaike fit error is also presented in the literature [28].

As it can be seen in this section, although significant work has been done in the field of renal image registration, there is much room for the development of validation strategies for renal MRI image registration. Due to missing agreement on a registration quality measurement, it is difficult to make a quantitative comparison between registration algorithms.

## Applications

Motion correction approaches in renal imaging are mainly applied to two imaging modalities, namely ASL and DCE-MRI. Most literature reviewed in the following thereby focuses on improving the imaging techniques and yet not report on directly on kidney diseases.

### Arterial spin labeling

It has been very well acknowledged that the ASL technique offers a great potential for noninvasive and exogenous contrast agent free renal perfusion quantification [4, 86]. Since it is a kind of differential blood perfusion measurement technique [87], applying it to kidney function characterization is particularly challenging. Even tag and control MR images taken within the same acquisition time-period may be spatially misaligned due to motion, and their difference may feature subtraction artifacts distorting the perfusion signal [88]. Moreover, as the acquired signal is small, multiple acquisitions are averaged to improve the signal-to-noise ratio which further increases the time difference between images of the same anatomical region and makes the images’ mismatch even worse. These effects call on the necessity of using effective methods of motion compensation to reduce artifacts and perfusion region blur. Those involve both acquisition-based and image post-processing-based approaches.

In a repeatability study of renal perfusion measurement using ASL [86], respiratory cycle triggering was used. This apparently extends the time of acquisition and solves the problem of motion partly. Nevertheless, the reduction of motion artifacts was achieved leading to repeatable quantification. The strategy of pace-breathing and breath holding was used in Gardener et al. with considerable reduction of the artifacts [41]. This, however, requires cooperation of the subject and might be impossible to accomplish in many cases.

The use of image background suppression (BGS) pulses to reduce unwanted image components, such as noise and motion artifacts, was proposed, e.g., by Garcia et al. [89] and Alsop et al. [90]. However, its contribution to an improvement of ASL quantification is currently debated [4].

An extensive investigation of the feasibility of background-suppressed renal ASL combined with retrospective image registration during the free-breathing acquisition is described in Bones et al. [91]. A hypothetical 100% suppression of the background signal would eliminate the signal of stationary tissue which might cause the registration task ill-defined [41, 92]. To cope with this effect, fat images can be acquired. As suggested in several papers [90, 91], fat tissue is suitable for providing the registration reference—it is characterized by short T1 time and thus recovers quickly from BGS pulses. The fat images for image registration were acquired in the same pseudo-continuous ASL (pCASL) acquisition and the effect of their use was compared to the use of ASL images themselves. Moreover, the background suppression was implemented at five different suppression levels, for qualitative and quantitative assessments of its influence on perfusion measurement quality. The ASL data sets were collected in relaxed, free-breathing conditions of ten healthy volunteers, using a 1.5 T MRI scanner equipped with 28-element phased-array receiver coil. Two strategies of image registration were implemented, separately for each kidney. In the first approach, ASL images of consecutive repetitions (*n*, e.g., *n* = 9) were co-registered with the tagged image of the first tag-control image pair. A 3D translation registration was implemented with the use of elastix software [93] using the Euler transform and b-spline interpolation. In a second approach, the consecutive fat images were co-registered with the first one and the correction results were transferred to respective ASL images. In addition, the equilibrium magnetization image M0, used to compute the perfusion-weighted images, was always registered to the fat image, to account for too large contrast difference in the case of background-suppressed ASL images. The visual inspection and quantitative results showed that background suppression increased precision without compromising accuracy of free-breathing ASL-based kidney perfusion measurement. This applies to both ASL image-based and fat image-based motion correction schemes. Finally, comparison was made with a paced-breathing acquisition leading to the conclusion that the proposed free-breathing technique with retrospective registration gives comparable perfusion estimation quality to this established but impractical method. Some parts of the implemented post-processing pipeline involve manual operations—setting the kidney ROI as an example. For instance, Bones et al. envision that machine learning algorithms might be a proper means to automate this task [91], but need further investigations.

The study by Nery et al. aimed at the development of a robust ASL-based technique for kidney perfusion measurement in pediatric subjects with CKD [38]. A single-shot background-suppressed 3D gradient- and spin-echo (GRASE) flow-sensitive alternating inversion recovery (FAIR) ASL acquisition method was implemented in a 1.5 T MRI scanner. Respiratory triggering was used to activate inversion pulses at end expiration. A separate proton-density (PD) image was acquired without any inversion or saturation pulses, for conversion of the perfusion-weighted signal into RBF. The RBF was quantified after retrospective image processing, including weighted averaging (to reduce the significance of corrupted ASL scans) and motion correction. The acquisition protocol was designed to ensure high SNR and robustness to motion. Two groups of subjects took part in the experiment. Five healthy adult volunteers of the first group were asked to remain still and breathe normally for the first ASL run, and then alter the amplitude and rate of their respiration—in the second run. The second group consisted of 11 children with severe CKD as indicated by the values of GFR. The effects of three post-processing options (no motion correction, image registration, and image registration combined with weighted averaging) were evaluated and compared across all data sets. All images in the saturation-recovery set were registered to the non-background-suppressed reference PD image. A mutual information similarity metric was used with stochastic-gradient-descent optimization, both features available in the elastix toolbox [93]. This study demonstrated quantitatively the importance of motion correction in reduction of artifacts in both T1 and RBF maps, and saturation-recovery fit errors, as well as increase of temporal signal-to-noise ratio (tSNR) of perfusion-weighted images and improve the repeatability of T1 and RBF measurements, especially in the pediatric subjects who featured high likelihood of kidney movements during MR scanning. The renal ASL was considered as a feasible method providing robust diagnostic information in case of pediatric subjects with severe kidney disease. The registration method was not characterized in more detail, e.g., suitability of other meta-parameter options available in elastics was not discussed.

Shirvani et al., showed recently the feasibility of multiparametric renal arterial spin labeling (3 T, 3D GRASE, FAIR, background suppression, and PD image acquired as the reference) with free-breathing acquisition [94]. The control and labeled images were motion-corrected by performing retrospective 2D elastic registration. Proprietary vendor software was used for this purpose, but neither background theory nor algorithm details governing its operation are presented in this paper. They refer to a work of Wu et al., where affine registration method was used, which could adjust for bulk motion; however, a non-linear elastic model may be more suited to the multi-TI ASL abdominal imaging [95]. Unfortunately, no discussion of this retrospective processing aspect is included in the work of Shirvani et al.

In Morra-Gutierrez et al., non-rigid diffeomorphic registration algorithm available in ANTs software [96] was employed, with the use of cross-correlation disparity metric to minimize residual motion [97]. A simultaneous segmentation-registration algorithm was applied to kidney motion correction by Hammon et al. [98]. Intentionally, rigid registration was implemented, as a means of evaluating registration errors and using it for sorting out acquisitions which do not show adequate quality. No details of the registration program used for kidney motion correction are described in Dong et al. [99]. A non-linear image registration implemented in Matlab was applied by Wang et al., again with no details on the underlying algorithm and similarity metrics [100]. Rigid registration with normalized mutual information metric was selected in study by Artz et al. [101], to apply ASL to native and transplanted kidneys. A non-rigid image registration with cross-correlation was chosen to register ASL image pairs in healthy volunteers undertaking different breathhold maneuvers in Schewzow et al. [46]. The significance of breathing strategies and background suppression is also discussed and illustrated in Robson et al. [8].

### DCE-MRI

Renal dynamic contrast-enhanced (DCE) MRI provides quantitative information on renal perfusion and filtration. Dynamic contrast-enhanced MRI (DCE-MRI) makes it possible to trace the circulation and distribution of injected low-molecular-weight contrast agents. It can be used to characterize microvascular structure and function in a developing tumor blood supply network. By fitting tracer kinetic models to DCE-MRI time series data, one can estimate the magnitude and spatial distribution of kinetic parameters, e.g., K^trans.^ [35]. For perfusion measurements to find widespread utility in the clinical environment, the exams must be easy to implement, robust to patient compliance issues such as problems with breath holding, and should be performed at clinically relevant resolutions with complete volumetric coverage [34].

It is believed that clinical implementation of this imaging technique is hampered by challenges in quantitative image analysis as a result of misalignment of the kidneys due to respiration and abdominal organ movements [80]. The latest articles on application of DCE-MRI to medical diagnosis are focused on assessing possibilities of free-breathing image acquisition and optimizing its processing pipeline. Both acquisition-based means and post-processing registration are considered.

In de Boer et al., automatic registration to fat images was performing best and allowed extraction of GFR estimates correlated with creatinine-based GFR values [80]. The authors claim that due to limited manual interaction, this method will be easy to implement in clinical practice.

An image post-processing framework was developed in Hanson et al. to study the significance of individual post-processing steps in a pipeline aimed at estimation of GFR from DCE images [102]. Twenty healthy volunteers underwent DCE-MRI examinations and serum biochemistry of Iohexol clearance for reference GFR measurements. In total, 692 different combinations of post-processing steps were explored for analysis. The application of classification trees and ensemble learning methods was useful for disclosing systematic patterns in the data that were not possible to detect by unaided reasoning and manual inspection.

The purpose of the study by Riffel et al. was to evaluate a technique for free-breathing dynamic contrast-enhanced renal magnetic resonance imaging (MRI) applying a combination of radial k-space sampling, parallel imaging, and compressed sensing [14]. There were no respiratory motion artifacts in any of the 25 patients, as investigated by two blinded radiologists. The renal plasma flow was estimated based on a volumetric analysis of the generated DCE perfusion maps, being close to physiologically expected value. The authors conclude that dynamic, motion-suppressed contrast-enhanced renal MRI can be performed in high diagnostic quality during free breathing using a combination of golden-angle radial sampling, parallel imaging, and compressed sensing.

To achieve high temporal and spatial resolution for renal DCE-MRI, fast imaging technique was used—3D through-time radial GRAPPA [34]. Despite high degree of undersampling, the images retained excellent quality. Ten patients were examined in free breathing and the images were registered to compensate for kidney motion. Non-linear Image Registration Tool (FNIRT) was used for that task [103]. Two-compartment model was applied for renal perfusion parameter estimation. An accurate high-resolution 3D quantitative renal functional mapping of perfusion and filtration parameters was obtained.

A combined registration–segmentation method was applied in Hodneland et al., for kidney motion correction in 4D DCE-MRI volumes [18]. The segmentation term affects the registration by enforcing time-course similarity of voxels. GFR values estimated with this technique show very little difference to Iohexol-measured GFR. The authors believe that segmentation-driven registration approach has a great potential for further development into pharmacokinetic GFR model-driven segmentation of the kidneys.

## Discussion/conclusion

The aim of this paper was to collect and review approaches to correct motion in renal MRI acquisitions. There is a large body of techniques ranging from acquisition-based motion suppression to post-processing-based image registration approaches, or a combination of the two. Besides them, simple breathhold strategies are used to mitigate organ motion and complement both mentioned approaches.

Results reported in the reviewed works state promising results; however, two points need to be considered in future to remove barriers in the development of renal MRI biomarkers by reducing errors introduced to motion artifacts in, e.g., renal perfusion imaging.

First, the papers do not provide sufficient implementation details of the registration to enable reproduction by other research groups. Although the objective functions used are usually outlined, some details are often missing, e.g., the size of Parzen windows when estimating joint probabilities or the interpolation methods used. The descriptions of the geometric transformation models lack the definition of used parameters, except of one paper only [10]. In other cases, details such as the number of B-spline control points or size of the displacement field smoothing kernel are not given. The description of search methods usually lacks details of the configuration of the multiresolution pyramids employed, e.g., number of resolutions, methods of transitions between resolutions, the approach to select the reference frame, or even selection of the optimization method used.

It is also noticed that papers often cite other papers where the employed methods are originally proposed and eventually well described, however, without solid reasoning of their selection or usage and setting. This makes the reproducibility of the published work most difficult and hinders broader usage and acceptance of the proposed methods. Eventually, a consensus is missing.

As a resource for image registration parameters, the elastix toolbox [93] provides a database of parameter settings [104] that allow for reproducing results. However, parameter settings dedicated for renal image registration are not reported yet. Towards consensus building in renal image registration attempts towards a parameter database, not necessarily based on a tool like elastix alone is needed.

A second important aspect of today’s research in (renal) image registration is that the evaluation is always based on presumptions and, thus, is unreliable. In most cases, it is based on the visual inspection of either registered images themselves or DCE time-courses.

In some work, also parameters such as perfusion or GFR calculated from the data are used to evaluate the image registration success, but could be seen as an intrinsic, relative evaluation metric, since the calculation of such parameters underlies addition sources of error [105]. In this, also good correlations of MR-based quantitative parameters with reference methods not using image registration were reported [106]. However, the authors point to image registration to further improve results as “3D data also suffered from significant intra- and inter-frame motion artifacts”.

In addition, synthetic data sets are reported, but, to some extent, are simplified. The only trustworthy way would be to use some reliable ground truth, eventually based on expert segmentations or markers. Not surprisingly, such ground truth seems nonexistent by today. As for the parametrization also for the evaluation, a database of ground truth data is needed to enable researchers to test and benchmark their techniques and in the long run to build a consensus on renal image registration.

The introduction of DL techniques into image registration enthuses with promised properties of speed and accuracy that have been long searched with traditional optimization approaches. However, DL relies on extraction of knowledge from training data sets, whose limited availability, especially in renal MRI, is already the major limitation to validation of registration algorithms and their clinical acceptance [6]. Registration methods in clinical environments must be able to cover diverse situations that include not only variability among healthy patients but also pathology cases, which adds an additional dimension to the required information content of the training databases. Even though the possibility of re-training and using digital phantoms may reduce required extent of the training databases considerably, availability of exhaustive enough training data sets is currently our primary concern regarding the clinical acceptability of DL methods.

Eventually, renal image registration approaches need to prove the additional benefit to the overall goal of estimating MR-based biomarkers to diagnose CKD. Today, this is yet not provided and needs further investigations. Thereby, the evaluation should take care of several aspects. First, the estimated parameters as the final outcome of such analysis pipeline are affected by errors in each step. The study of Hanson et al., yet the only covering this topic, nicely outlines such procedure [102]. Second, these errors, e.g., introduced by pharmacokinetic modeling might possibly render larger than those by motion corruption. Especially, this might be the case when a whole kidney ROI analysis is performed as the data are usually smoothed over the ROI and motion is, therefore, of minor impact. Respective studies for instance report comparable parameter estimates to gold standard techniques [106]. However, a benefit of MRI-based perfusion analysis is to be able to provide perfusion measurements on voxel-wise basis allowing to capture tissue perfusion heterogeneity. Since kidney motion is usually larger than the voxel size (up to 86 mm are reported [42]), image registration seems, therefore, reasonable as reported by several studies so far [14, 25, 37].

This review shows that although there are some open issues mainly related to evaluation, image registration algorithms are being adapted to renal MRI and already contribute to the renal analysis. In addition to traditional image registration methods, a new category of DL methods is also already emerging in the renal MRI. Workgroup two of the PARENCHIMA COST action CA16103 works towards increasing availability of renal MRI data and processing algorithms for wider acceptance of renal MRI biomarkers in research and clinical practice. This includes collecting image database necessary for method development and validation, reaching consensus on evaluation strategies and establishing a database of evaluated algorithms. In this review, we identified limitations and uncertainties as well as prospects of renal MRI registration methods and applications, in relation to papers published on the subject. We expect that a significant progress will be made in the field, regarding reliability, accuracy, and processing speed. This will contribute to more objective and accurate personalized diagnosis of kidney diseases.
