# Correction to: Image registration in dynamic renal MRI—current status and prospects

**DOI:** 10.1007/s10334-020-00850-8

**Published:** 2020-06-11

**Authors:** Frank G. Zöllner, Amira Šerifović-Trbalić, Gordian Kabelitz, Marek Kociński, Andrzej Materka, Peter Rogelj

**Affiliations:** 1 Computer Assisted Clinical Medicine, Medical Faculty Mannheim, Heidelberg University, Theodor-Kutzer-Ufer 1-3, 68167 Mannheim, Germany; 2 Faculty of Electrical Engineering, University of Tuzla, Tuzla, Bosnia and Herzegovina; 3 Institute of Electronics, Lodz University of Technology, Lodz, Poland; 4 Faculty of Mathematics, Natural Sciences and Information Technologies, University of Primorska, Koper, Slovenia

## Correction to: Magnetic Resonance Materials in Physics, Biology and Medicine (2020) 33:33–48 10.1007/s10334-019-00782-y

The article Image registration in dynamic renal MRI—current status and prospects, written by Frank G. Zöllner, Amira Šerifović‑Trbalić, Gordian Kabelitz, Marek Kociński, Andrzej Materka and Peter Rogelj, was originally published electronically on the publisher’s internet portal on 9 October 2019 without open access.With the author(s)’ decision to opt for Open Choice the copyright of the article changed on 24 April 2020 to © The Author(s) 2020 and the article is forthwith distributed under a Creative Commons Attribution 4.0 International License (https://creativecommons.org/licenses/by/4.0/), which permits use, sharing, adaptation, distribution and reproduction in any medium or format, as long as you give appropriate credit to the original author(s) and the source, provide a link to the Creative Commons licence, and indicate if changes were made.

The original article has been corrected.

